# Posterior Transpedicular Dynamic Stabilization versus Total Disc Replacement in the Treatment of Lumbar Painful Degenerative Disc Disease: A Comparison of Clinical Results

**DOI:** 10.1155/2013/874090

**Published:** 2013-01-17

**Authors:** Tunc Oktenoglu, Ali Fahir Ozer, Mehdi Sasani, Yaprak Ataker, Cengiz Gomleksiz, Irfan Celebi

**Affiliations:** ^1^Neurosurgery Department, American Hospital, 34365 Istanbul, Turkey; ^2^Neurosurgery Department, Koc University School of Medicine, 34450 Istanbul, Turkey; ^3^Physical Therapy and Rehabilitation Department, American Hospital, 34365 Istanbul, Turkey; ^4^Neurosurgery Department, Mengücek Gazi Training and Research Hospital,School of Medicine, Erzincan University, 2400 Erzincan, Turkey; ^5^Radiology Department, Sisli Etfal Hospital, 34360 Istanbul, Turkey

## Abstract

*Study Design*. Prospective clinical study. *Objective*. This study compares the clinical results of anterior lumbar total disc replacement and posterior transpedicular dynamic stabilization in the treatment of degenerative disc disease. *Summary and Background Data*. Over the last two decades, both techniques have emerged as alternative treatment options to fusion surgery. *Methods*. This study was conducted between 2004 and 2010 with a total of 50 patients (25 in each group). The mean age of the patients in total disc prosthesis group was 37,32 years. The mean age of the patients in posterior dynamic transpedicular stabilization was 43,08. Clinical (VAS and Oswestry) and radiological evaluations (lumbar lordosis and segmental lordosis angles) of the patients were carried out prior to the operation and 3, 12, and 24 months after the operation. We compared the average duration of surgery, blood loss during the surgery and the length of hospital stay of both groups. *Results*. Both techniques offered significant improvements in clinical parameters. There was no significant change in radiologic evaluations after the surgery for both techniques. *Conclusion*. Both dynamic systems provided spine stability. However, the posterior dynamic system had a slight advantage over anterior disc prosthesis because of its convenient application and fewer possible complications.

## 1. Introduction 

Currently, one of the most important causes of chronic low back pain is thought to be a painful disc [1–3]. Some biomechanical and biochemical changes play a role in intervertebral disc degeneration; on the other hand intrinsic, extrinsic, and genetic factors are also important. Compression of the spine, torsional injuries, overload, and congenital anomalies have been shown to contribute to disc degeneration with applying excessive pressure onto intervertebral discs [4–10]. Despite numerous research studies, the etiology and physiopathology of disc degeneration remain unknown [2]. Annular tears resulting from degeneration of the annulus fibrosis, that contains pain receptors and internal disc ruptures, are the most common cause of pain [11–13]. Today, it is believed that degenerative disc disease (DDD) might cause instability in spine segments, and it is widely accepted that progressive back pain results due to this instability [14–16]. In fact, segmental instability begins when disc height deterioration is initiated by the progression of intervertebral disc degeneration. Instability as a consequence of disc degeneration has been described by Frymoyer [14, 15] as primary segmental instability and by Kirkaldy-Willis and Farfan [2] as the discogenic pain and instability stage in the overall process of degeneration. Benzel [16] included degenerative disc disease among the chronic instabilities and described the disease as “dysfunctional segmental motion” and “torsional instability.” Fusion is the standard surgical treatment option for painful lumbar degenerative disc disease that is unresponsive to conservative treatment modalities. Nonetheless, the side effects of fusion (pseudarthrosis, adjacent segment disease, and the donor site morbidity) and suboptimal clinical satisfaction rates, which have been reported even in patients with radiologically observed fusion, have led to a search for alternative treatments [17–22]. 

Numerous dynamic techniques were developed over the last two decades. Recently, these devices were classified as total disc replacement (TDR) and posterior transpedicular dynamic systems (PTDS) [23]. Both PTDS and TDR have been widely used in surgical treatment of degenerative disc diseases of the lumbar spine. Numerous studies showed promising clinical results [24–35]. However, there is no study that compares the TDR and PTDS techniques in the treatment of DDD.

In this prospective study, we evaluated and compared the clinic and radiologic outcome of TDR and PTDS in patients with painful lumbar degenerative disc disease through an extensive literature review. 

## 2. Material and Methods

### 2.1. Total Disc Replacement Group

We performed TDR on 25 patients (14 females and 11 males). The mean age of the patients was 37.32 (with a range from 25 to 50), and the mean follow-up period was 29.16 months (with a range from 24 to 42 months).

A lumbar total disc replacement (Maverick, Medtronic Sofamor Danek, Memphis, TN, USA) was placed into the intervertebral disc space with open window laparotomy technique [36]. 

**Figure 1:**
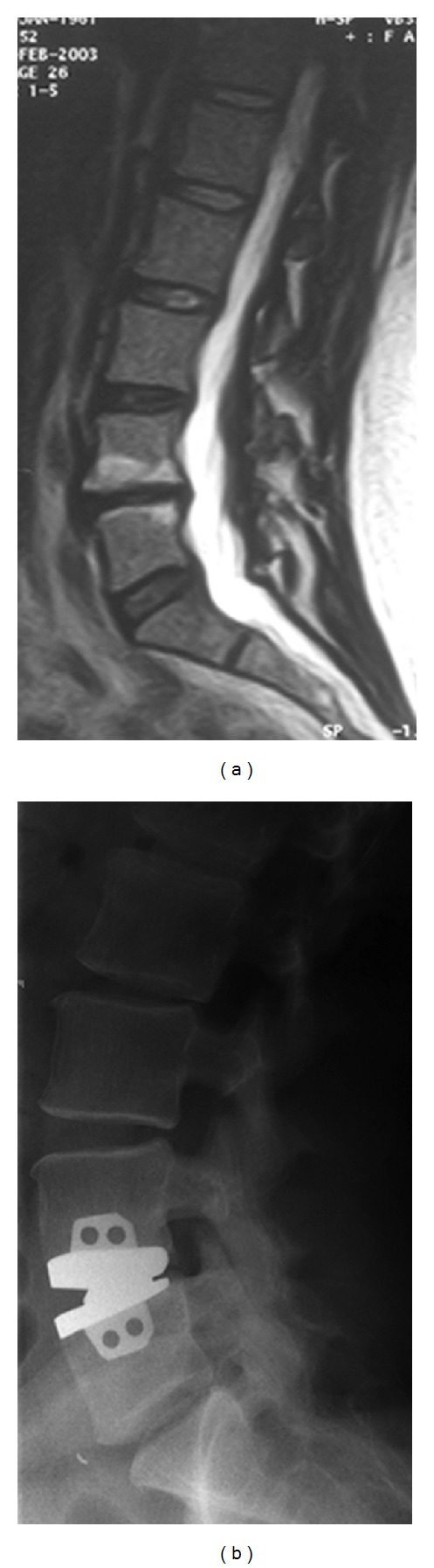
A 30-year-old woman complained of severe back pain attacks. She had no neurological deficits. (a) T2-weighted MRI scans showed advanced degeneration with Modic changes in the L4-L5 disc. (b) Maverick disc prosthesis was applied.

All patients in the TDR group had a lumbar single-level painful disc. 15 patients showed L4-L5 DDD, and 10 patients showed L5-S1 DDD (Figure 1). All of the patients were informed about the surgery, and they signed a written, informed consent form. The inclusion criteria for TDR surgery included a complaint of lower back pain that had duration of at least 12 months and at least six months of conservative treatment without satisfactory results. Other inclusion criteria were that the patients must be less than 50 years old and have no signs of lumbar degenerative spondylolisthesis or osteoarthritis in their facet joints, which was confirmed with computerized tomography (CT) and dynamic plain radiographs. The patients also had to have symptomatic lumbar degenerative disc disease that was visible in magnetic resonance imaging (MRI) as a blackened disc as well as a confirmation of the diagnosis by displaying pain behaviors during discography. 

### 2.2. Posterior Dynamic Transpedicular Stabilization Group 

We performed posterior dynamic transpedicular stabilization on 25 patients (13 females and 12 males). The mean age of the patients was 43.08 years (with a range from 24 to 55 years), and the mean follow-up period was 36.48 months (with a range from 24 to 48 months). 

Patients in the dynamic posterior stabilization group were operated with the Cosmic (Ulrich GmbH & Co. KG, Ulm, Germany) posterior dynamic transpedicular stabilization system (hinged screw-rigid rod) through transmuscular approach [35]. 

**Figure 2:**
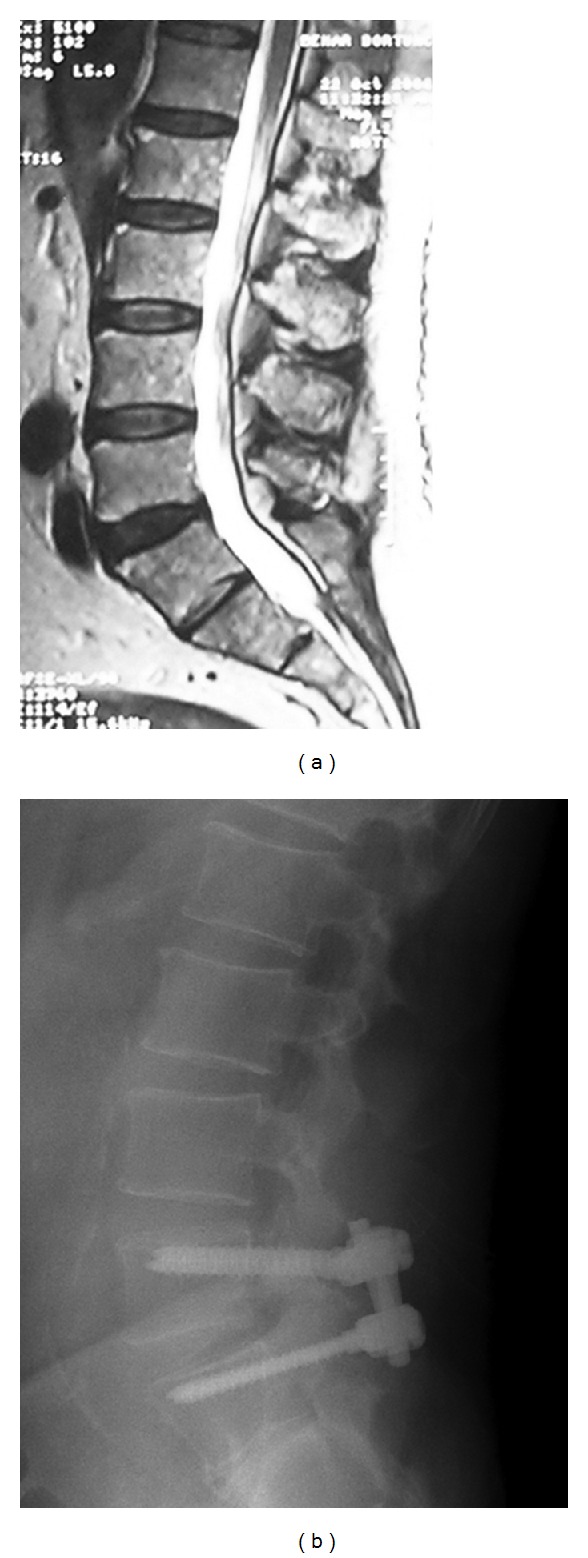
(a) A 50-year-old male complained of severe back pain attacks. (a) T2-weighted MRI scans showed degeneration of the disc at L4-L5 lumbar disc (L5- S1 considered as sacralization). (b) Following posterior transpedicular dynamic stabilization with the Cosmic system.

All cases in the PTDS group had one-level painful disc disease. The operated discs were L4-L5 region (16 cases) and L5-S1 region (9 cases) (Figure 2). 

Similar to the patients in the TDR group, the inclusion criteria included a confirmed diagnosis of symptomatic lumbar degenerative disc disease through MRI and positive discography, a complaint of lower back pain that had a duration of at least 12 months, at least 6 months of conservative treatment without satisfactory results, and the absence of apparent instability confirmed with lumbosacral dynamic X-rays.

### 2.3. Clinical Evaluation

We evaluated and compared the average surgical time, blood loss during the surgery, and the length of the stay in hospital for both groups of patients (Table 1). The visual analog scale (VAS) and the Oswestry Disability Index (ODI) were used for the clinical evaluations and follow-up examinations. Clinical evaluations of the patients were carried out in the data at preoperative period and 3, 12, and 24 months after the surgery (Tables 2 and 3). 

### 2.4. Radiological Evaluation

**Figure 3:**
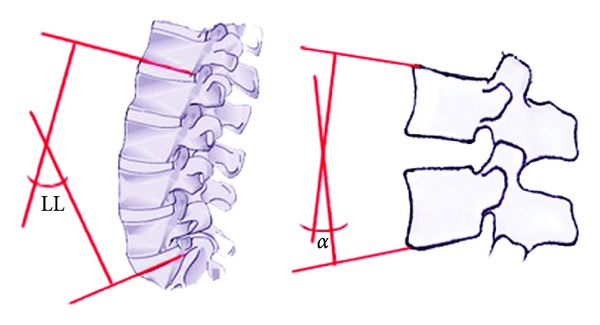
Lordosis of the lumbar spine (L1-S1) was measured via the angle between the lines drawn from the lower endplate of L1 and the upper endplate of S1 (LL). Additionally segmental lordosis (*α*) at the operation level was measured via the angle between the lines drawn from the upper and lower endplates of the vertebrae that form the operation segments.

To diagnose lumbar disc disease, an MRI examination of each patient was performed and a black disc was observed. Pain symptoms were confirmed with the detection of provocative pain through a discography which was applied to the black disc. Lumbosacral plain and dynamic (hyperflexion and hyperextension) X-rays and CT examinations of the patients were carried out by independent radiology experts in preoperative. Follow-up plain X-Ray studies were obtained 3, 12, and 24 months after the surgery. Control CT study was performed in postoperative 24 months. Loose screws as well as broken screws, instrument migration, subsidence, and spontaneous fusion were evaluated. Additionally lumbar lordosis angle (LL) and segmental lordosis angle (*α*) data was obtained (Tables 4 and 5) (Figure 3).

### 2.5. Statistical Analysis

The Number Cruncher Statistical System (NCSS) 2007 and 2008 PASS Statistical Software (Utah, USA) were used for statistical analysis of the data. In addition to descriptive statistical methods (e.g., mean, standard deviation), Student's *t*-test was used to compare the normally distributed parameters between the two groups. The Mann-Whitney *U* test was used for the comparison of parameters with nonnormal distribution. Bonferroni test was used to compare the follow-up data with normal distribution, and paired sample *t*-test was used for dual comparison. In nonnormal distribution group, the follow-up data compared with Friedman test and Wilcoxon test was used for dual comparison. The significance level was *P* < 0.05.

## 3. Results

There was statistically significant difference observed between the mean ages and follow-up periods of the groups (*P* < 0.05) (Table 1). The PTDS applied to significantly older patients was compared to TDR group. 

**Figure 4:**
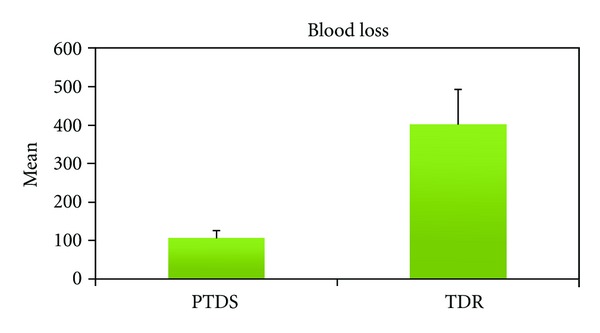
The data of blood loss in surgery for both groups.

There was a statistically significant difference (*P* < 0.01) between the level of blood loss in the two groups. The level of blood loss was significantly higher in the TDR group compared to the PTDS group (Table 1, Figure 4). 

**Figure 5:**
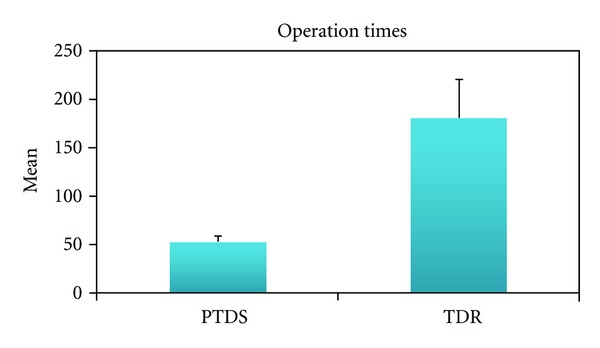
The data of operation times for both groups.

The operation time was significantly longer (*P* < 0.01) in the TDR group compared to the posterior dynamic stabilization group (Table 1, Figure 5).

There was significant difference in the length of the hospital stay between the two groups (*P* < 0.05) (Table 1). 

Preoperative VAS and ODI levels were not significantly (*P* > 0.05 and *P* > 0.05) different between the groups (Tables 2 and 3).

**Figure 6:**
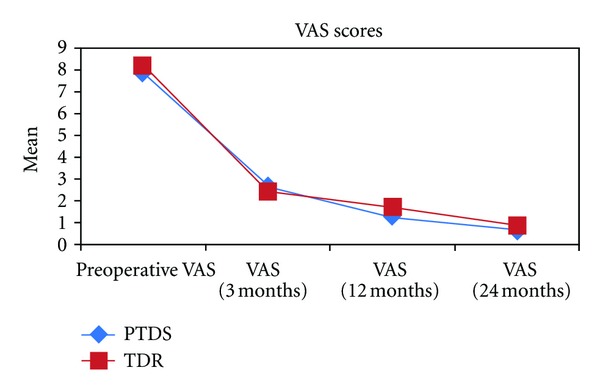
The distribution of VAS scores for both groups.

**Figure 7:**
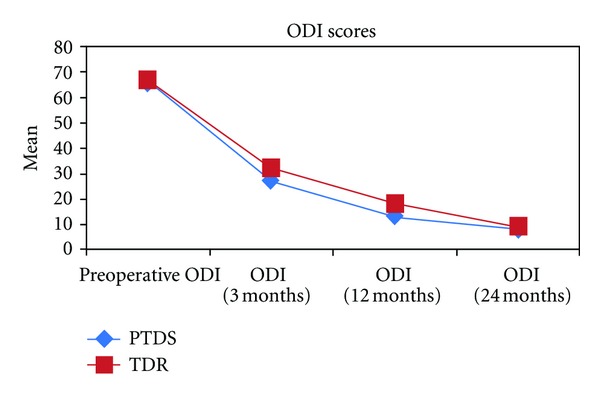
The distribution of ODI scores for both groups.

In both groups the clinical parameters (VAS and ODI) showed significant improvement in all postoperative time periods when compared to preoperative data (Tables 2 and 3, *P* < 0.01). There were no statistically significant differences observed between the groups for the each follow-up VAS (*P* < 0.05, Table 2). The ODI data showed significant difference only in postop 12 months. The PTDS group had significantly better outcome in this time period. However this advantage did not persist. There was no significant difference in 24-month scores (*P* > 0.05, Table 3) (Figures 6 and 7). 

There were no significant differences observed between the preoperative and postoperative lumbar (LL) and segmental lordosis (alpha) evaluations for both techniques (*P* > 0.05) (Tables 4 and 5). 

No surgical morbidity and/or complications observed in the group treated with PTDS. There were two iliac vein injuries that occurred in two patients in the TDR group. These injuries were sutured in the operation with no mortality and residual morbidity.

## 4. Discussion

Fusion has been widely used as a surgical treatment for painful disc disease. Fusion eliminates the abnormal movements and offers satisfactory outcome. On the other hand, even in patients with 100% fusion achieved with applying 360° fusion method, the satisfaction rate is not necessary optimal and might be low as 30% [19–21, 23, 24]. Donor site problems have also been a significant complication in fusion surgery [20]. Therefore, alternative treatment techniques were developed in an attempt to prevent side effects that are commonly observed after fusion surgery and to improve the patient satisfaction rate. In recent years, dynamic systems that provide spine mobility have been developed to avoid the well-known side effects of fusion technique. Today indications and contraindications of TDR and PTDS are well known [23]. Both techniques can be used for the same indications. A painful black disc can be treated with application of either technique. 

TDR was developed over the past ten years as a promising surgery that was preferable over fusion surgery because the proponents of TDR claimed that the procedure preserves mobility and reduces the risk of adjacent segment disease. After a ten-year effort by Büttner-Janz et al., TDR was announced as a new solution method for painful disc disease [37]. Numerous TDR systems were developed and offered for clinical application [29, 32, 38]. Biomechanical studies showed that the TDR prosthesis stabilizes the spine while providing nearly intact segmental motion [29, 32, 39]. Early clinical results of TDR in the treatment of DDD showed promising outcomes [37, 40–46]. 

The patients treated with TDR usually have short recuperation times and less postoperative pain compared to fusion procedure. On the other hand, TDR application has several significant limitations including; (a) the patient should be between 30 and 50 years old, (b) there should not be any posterior column disruption, (c) intervertebral disc height should be ≥4 mm, and (d) single-level DDD is more appropriate to apply TDR. Beside these limitations TDR is an anterior approach which has its inherent risks such as injury to intra-abdominal organs and vascular structures. Additionally, the lesions in the peritoneal cavity caused by abrasion, ischemia, desiccation, infection, thermal injury, and foreign bodies can result in adhesion formation [47].

TDR is used extensively around the world; however severe complications have been associated with the technique [48, 49]. In this study we observed mild iliac vein injuries during the placement of the lumbar disc prosthesis in the two patients within the TDR group. Other possible disadvantages of TDR technique are as follows: revision surgery is quite difficult, biomechanically the L5-S1 level had no normal segmental motion, and results of two-level TDR use were not considered to be satisfactory according to patients [50]. Putzier et al. [51] concluded that the long-term results of a study by Charité were not satisfactory and they concluded their article yearning to fusion technique. 

Guyer et al. [52] published the results of a 5-year study showing that TDR was not superior to fusion. The authors concluded that there was no strong evidence that TDR was superior to fusion, and they suggested that high-quality, randomized controlled trials with relevant control groups and a long-term followup were needed to evaluate the effectiveness and safety of TDR [53]. 

Posterior dynamic stabilization systems are designed to increase the success of spinal surgery and to eliminate the complications of fusion with rigid instrumentation such as adjacent segment disease (12.2–18.5%) [54] due to the stress-shielding properties (2–3% per year after stabilization) [55], pseudarthrosis (3–55%) [18, 56, 57], device-related osteopenia [58], and loss of motion in fused spinal segments. Besides these side effects of fusion, clinical healing might be suboptimal in cases even with satisfactory radiological results [22, 59]. Therefore, the use of posterior dynamic stabilization in the surgical treatment of DDD may provide greater patient satisfaction, resulting from shorter hospital stays, less recuperation time, and none of the disadvantages related to fusion, which requires more invasive procedures.

Numerous biomechanical studies proved that hinged screw stabilization can stabilize the spine almost as well as the rigid screw stabilization used in fusion surgery in the treatment of chronic lumbar instability [60, 61]. There are no randomized controlled studies in the literature because PTDS is a new technique. However, there are many retrospective studies that are precursors for future randomized studies. Recently, studies on PTDS have shown very encouraging clinical results and demonstrated that these systems provide stabilization that is similar to the posterior rigid stabilization obtained with fusion surgery [23–28, 30, 31, 34, 35, 60, 62, 63].There are few studies which concluded that dynamic stabilization is not superior to rigid stabilization [63–66]. Although these results showed that there was no advantage of PTDS over fusion surgery in clinical outcome, on the other hand these studies also showed that PTDS is superior to fusion due to the simplicity of the procedure, low morbidity, and reduced hospitalization time to achieve similar satisfactory outcome as fusion. Similarly numerous studies have shown that posterior dynamic transpedicular stabilization caused less intraoperative blood loss and had a shorter operating time [25, 28, 31, 35]. Furthermore, several studies reported that PTDS slows down intervertebral disc degeneration by removing the load from the degenerative disc tissue and providing better load distribution which is an important advantage of this technique [25, 27, 30]. 

Based on previous studies, if a disc is in the beginning stage of degeneration and if there is only posterior annulus defect, the disc might repair itself after PTDS. On the other hand, if the disc has advanced degeneration including decreased disc height, significant dehydration, and/or slight bulging, fusion might occur slowly after PTDS. However, in both of these scenarios, the patient would be pain-free. In cases of advanced disc degeneration, the fusion results are satisfactory because fusion occurs easily. If PTDS is applied to advanced disc degeneration cases, the segments might fuse and the results will be the same. Therefore in regard of motion preservation, TDR may be a superior treatment in this group of patients if they have intact facet joints. 

Huang et al. [67] reported the advantages and disadvantages of nonfusion technology in spinal surgery. Some of the potential benefits of nonfusion implants were the elimination of possible complications due to bone grafts and pseudarthrosis as well as a reduction in the surgical morbidity and the incidence of adjacent level degeneration. The potential risks of nonfusion implants included mechanical failure, dissolution and migration, subsidence, and same-level degeneration.

Previous studies suggested that lumbar total disc prosthesis would reduce the stress on the adjacent disc, after sagittal balance is restored. Harrop et al. [68] reviewed the literature on lumbar adjacent segment degeneration after fusion and TDR. They concluded that adjacent segment disease had a stronger relationship with fusion than arthroplasty. Stoll et al. [34] reported symptomatic adjacent segment disease in 9% of their posterior dynamic transpedicular stabilization patients after a 38-month follow-up period. Cakir et al. [63] reported their results after performing PTDS with Dynesys and TDR with ProDisc (Synthes-Spine Solutions, New York, NY). They suggested that both dynamic systems were promising alternative options compared to fusion for patients with different pathologies because of reduced morbidity. Cakir et al. [63] obtained good clinical results with both systems. Both TDR and PTDS result in less adjacent segment disease. Although a reduced incidence of adjacent segment degeneration appears to be the most important advantage of nonfusion systems, this advantage has not been proven.

Considering all of the features of both techniques, PTDS is a less invasive surgery compared to fusion and TDR techniques. Additionally, PTDS has no age limitation and does not require intact posterior spinal column as TDR technique. Finally, anterior lumbar disc prosthesis requires transperitoneal or retroperitoneal intervention and usually requires a multidisciplinary approach (general surgeon, cardiovascular surgeon, and spinal surgeon). Naturally, the complication rate decreases with a conventional surgical approach and increases when complex anatomical structures are involved in the surgery.

## 5. Conclusion

In this study, we observed that both dynamic techniques TDR and PTDS offered satisfactory outcome in the surgical treatment of lumbar DDD. However, in this limited study, PTDS had several advantages over TDR such as (a) less invasive technique, (b) shorter operation time, (c) less intraoperative bleeding, and (d) lower complication rates. Further prospective, randomized clinical studies with a larger number of patients and with a longer follow-up period are needed to support our findings. 

## Figures and Tables

**Table 1:** Comparison of TDR and PTDS groups.

		TDR (*n* = 25)	PTDS (*n* = 25)	*P*
Age	Min–Max	25–50	24–55	0.018*
	Mean ± SD	37.32 ± 6.62	43.08 ± 9.65	
Followup (month)	Min–Max	24–42	24–48	0.001**
	Mean ± SD	29.16 ± 4.77	36.48 ± 6.72	
Length of hospital stay (day)	Min–Max	3–5	2–5	0.022*
	Mean ± SD	3.56 ± 0.58	3.04 ± 0.93	
Operation time (minute)	Min–Max	120–260	40–70	0.001**
	Mean ± SD	181.20 ± 39.40	52.40 ± 7.79	
Blood loss (mL.)	Min–Max	300–600	75–175	0.001**
	Mean ± SD	402.00 ± 91.83	103.00 ± 22.03	

Student's *t*-test, *P < 0.05, **P < 0.01.

**Table:** (a)

VAS		TDR (*n* = 25)	PTDS (*n* = 25)	^ a^ *P*
Preop VAS	Min–Max	6–10	6–9	0.219
	Mean ± SD	8.24 ± 1.09[8]	7.96 ± 0.79 [8]	
VAS (3 months)	Min–Max	0–5	1–5	0.588
	Mean ± SD	2.44 ± 1.16 [2]^‡^	2.64 ± 0.91 [2]^‡^	
VAS (12 months)	Min–Max	0–3	0–4	0.087
	Mean ± SD	1.68 ± 0.85 [2]^‡^	1.28 ± 0.94 [1]^‡^	
VAS (24 months)	Min–Max	0–2	0–3	0.240
	Mean ± SD	0.84 ± 0.69 [1]^‡^	0.68 ± 0.85 [1]^‡^	

	^ b^ *P*	0.001**	0.001**	

^a^Mann-Whitney *U* test, ^b^Friedman test.

^‡^Wilcoxon signed-rank test *P* < 0.001.

**P < 0.01.

**Table:** (b)

VAS (decrease %)	TDR (*n* = 25)	PTDS (*n* = 25)	*P*
	Mean ± SD	Mean ± SD	
Preop ∗ VAS (3 months)	70.18 ± 14.14	66.18 ± 13.57	0.374
Preop ∗ VAS (12 months)	79.91 ± 10.00	83.71 ± 12.08	0.214
Preop ∗ VAS (24 months)	89.86 ± 8.41	91.30 ± 10.78	0.519

Mann-Whitney *U* test.

**Table:** (a)

ODI		TDR (*n* = 25)	PTDS (*n* = 25)	^ a^ *P*
Preop ODI	Min–Max	40–100	46–98	0.847
	Mean ± SD	67.20 ± 20.79	66.16 ± 16.82	
ODI (3 months)	Min–Max	12–56	12–38	0.069
	Mean ± SD	32.32 ± 10.87^‡^	27.16 ± 8.63^‡^	
ODI (12 months)	Min–Max	4–34	2–26	0.010*
	Mean ± SD	18.00 ± 7.64^‡^	12.88 ± 5.75^‡^	
ODI (24 months)	Min–Max	2–20	2–18	0.408
	Mean ± SD	9.12 ± 4.28^‡^	8.04 ± 4.53^‡^	

	^ b^ *P*	0.001**	0.001**	

^a^Student's *t*-test, ^b^repeated measures test.

^‡^Adjustment for multiple comparisons: Bonferroni *P* < 0.01.

*P < 0.05, **P < 0.01.

**Table:** (b)

ODI (decrease %)	TDR (*n* = 25)	PTDS (*n* = 25)	*P*
	Mean ± SD	Mean ± SD	
Preop ∗ ODI (3 months)	49.96 ± 16.46	54.91 ± 20.00	0.669
Preop ∗ ODI (12 months)	72.80 ± 9.33	78.22 ± 11.68	0.093
Preop ∗ ODI (24 months)	86.15 ± 6.15	86.33 ± 8.92	0.808

Mann-Whitney *U* test.

**Table 4:** The comparison of LL angles.

LL		TDR (*n* = 25)	PTDS (*n* = 25)	^ a^ *P*
Preop LL	Min–Max	25–65	34–72	0.948
	Mean ± SD	49.60 ± 10.46	49.80 ± 11.26	
LL (3 months)	Min–Max	26–65	34–69	0.747
	Mean ± SD	49.52 ± 9.51	48.60 ± 10.52	
LL (12 months)	Min–Max	24–64	30–67	0.764
	Mean ± SD	49.60 ± 10.15	48.72 ± 10.50	
LL (24 months)	Min–Max	22–65	35–65	0.786
	Mean ± SD	49.56 ± 10.38	48.80 ± 9.30	

	^ b^ *P*	0.998	0.890	

^a^Student's *t*-test, ^b^repeated measures test.

LL: lumbar lordosis.

**Table 5:** The comparison of SL (*α*) angles.

ALPHA		TDR (*n* = 25)	PTDS (*n* = 25)	^ a^ *P*
Preop ALPHA	Min–Max	4–17	4–30	0.274
	Mean ± SD	10.32 ± 3.06	11.68 ± 5.33	
ALPHA (3 months)	Min–Max	3–19	3–33	0.566
	Mean ± SD	10.40 ± 3.70	11.20 ± 5.84	
ALPHA (12 months)	Min–Max	4–16	2–31	0.392
	Mean ± SD	10.36 ± 2.90	11.52 ± 6.05	
ALPHA (24 months)	Min–Max	5–14	3–30	0.248
	Mean ± SD	10.32 ± 2.28	11.56 ± 4.79	

	^ b^ *P*	0.989	0.858	

^a^Student's *t*-test, ^b^repeated measures test.

SL: segmental lordosis.
